# Effects of cinnamon essential oil on the physiological metabolism of *Salmonella enteritidis*

**DOI:** 10.3389/fmicb.2022.1035894

**Published:** 2022-12-06

**Authors:** Zhen Zhang, Yuanyuan Zhao, Xueqin Chen, Wei Li, Li Wang, Wen Li, Jianming Du, Shengxiang Zhang

**Affiliations:** College of Food Science and Engineering, Gansu Agricultural University, Lanzhou, China

**Keywords:** cinnamon essential oil, *Salmonella enteritidis*, physiological metabolism, new natural food preservatives, antibacterial mechanism

## Abstract

Food safety and health are the themes of today's society. As a class of foodborne pathogens, *Salmonella enteritidis* has become one of the common zoonotic pathogens. Because chemical preservatives have certain harmfulness and have been questioned, it is particularly important to find green and safe natural preservatives. The advantages of plant essential oils (EOs) are that they are green and safe, have a wide range of antibacterials, and are not easy to form drug resistance. In recent years, studies have found that EOs have excellent antibacterial activity, but their antibacterial mechanism has not been conclusive, which has certain limitations in their application in the food field. Cinnamon essential oil (CEO) extracted from dried cinnamon is a secondary metabolite of cells and a very important natural food flavor. More importantly, it is non-toxic to the human body and has been proven to have a good antibacterial effect, but its antibacterial mechanism is still unclear. Therefore, it was of great practical significance to carry out the research on the antibacterial mechanism of CEO on *S. enteritidis*. In this work, *S. enteritidis* was used as the test bacteria, and CEO was selected as the antibacterial agent to study the antibacterial mechanisms. By studying the physiological metabolism of *S. enteritidis* cells by CEO, the influence of CEO on the bacteriostatic mechanism of *S. enteritidis* was systematically elucidated. The study found that CEO treatment would reduce the activity of bacterial metabolism. It is mainly reflected in the following three aspects: first, the activity of key enzymes in TCA circulation is inhibited, thus affecting the respiration of *S. enteritidis*. Second, it affects the level of energy metabolism by inhibiting the content of adenosine triphosphate (ATP) and the activity of ATPase. Finally, it can affect the physiological metabolism of bacteria by inhibiting the metabolism of proteins and other substances. Therefore, this article was expected to provide a theoretical basis for the development of new natural food preservatives and the prevention and control of *S. enteritidis*.

## Introduction

With the continuous increase of the world's population, the demand for food types has gradually increased, and more and more food safety issues have been brought to the forefront. Therefore, food safety issues have become one of the important challenges that today's society faces. The World Health Organization report showed that *Salmonella* was at the forefront of foodborne pathogens, and *salmonella* contamination of varying degrees exists in most parts of the world, which seriously threatens public health and causes serious economic losses (Chousalkar et al., 2018). *Salmonella* belongs to the family enterobacteriaceae and is a Gram-negative microorganism with flagella, no spores, and turns red after staining. Most *Salmonella* species cause food poisoning through food transmission, of which animal foods account for the majority (Lamas et al., 2018).

Although chemical preservatives have been widely developed and used, they have caused great harm to human health. Therefore, it was of great significance to choose and develop green and natural antibacterial substances. Among many antibacterial agents, EOs have attracted the attention of scholars due to their advantages of green safety, broad-spectrum sterilization, and drug resistance. At present, spice essential oils have been widely used in the food preservative industry, and there are a large number of essential oil plant resources in China, which are very beneficial for the development and utilization of EOs.

At present, spice EOs have been widely used in food preservation and preservation, and their components are complex (Do Nascimento et al., 2020). By studying the physiological metabolism of CEO on *S. enteritidis* cells, the influence of CEO on the bacteriostatic mechanism of *S. enteritidis* is systematically clarified. The specific mechanisms are as follows: CEO treatment would reduce bacterial metabolic activity. It is mainly reflected in the inhibition of key enzyme activities in the TCA cycle, which affects the respiration of *S. enteritidis*. It affects the level of energy metabolism of the body by inhibiting the content of adenosine triphosphate (ATP) and the activity of ATPase. It can also affect the physiological metabolism of bacteria by inhibiting the metabolism of proteins and other substances. Therefore, this article is expected to provide a theoretical basis for the development of new natural food preservatives and the prevention and treatment of enteritis. Studies have shown that EOs have obvious inhibitory effects on important foodborne pathogens such as *Salmonella* (Guillin et al., 2021; Valdivieso-Ugarte et al., 2021). Cinnamon was a dicotyledonous lauraceae plant, and its bark, leaves, and other tissues contain a large amount of volatile oils, flavanols, polyphenols, coumarin, lignin, flavonoids, etc. (Yang Y.L. et al., 2021). Cinnamon essential oil (CEO) is extracted from cinnamon bark, cinnamon leaves, etc. It is a yellow oily liquid, non-toxic, and volatile. Research has shown that the main component of CEO is cinnamaldehyde (Chen et al., 2021; Modi et al., 2021). CEO is a broad-spectrum antibacterial agent that can kill *Pseudomonas aeruginosa* at low concentrations (Elcocks et al., 2020). Maggio et al. (2022) studies found that lily essential oil has good potential to alter the growth of *Listeria monocytogenes* in the food environment, an effect that may hinder the growth of pathogenic bacteria and contribute to food safety (Maggio et al., 2022). Somrani et al. (2020) studies demonstrated that CEO can effectively inhibit the attachment of *Listeria monocytogenes* naive cells on biofilms (Somrani et al., 2020). At the same time, it was found that CEO can effectively inhibit the growth of *Listeria monocytogenes* and *Salmonella typhimurium* in milk (Mortazavi and Aliakbarlu, 2019). Jeong et al. found that CEO can also inhibit the growth of oral caries-causing acidic bacteria (Jeong et al., 2021). CEO can disrupt the cell membrane integrity of *Clostridium oxysporum*, the main pathogen causing “Red Yang” kiwifruit anthracnose (He et al., 2018). Elcocks et al. found that CEO can rapidly kill *Pseudomonas aeruginosa* by affecting its cell membrane permeability and integrity (Elcocks et al., 2020). However, there are few studies on the effect of CEO on the mechanism of salmonella cell damage.

Therefore, in this study, CEO was used as a natural antibacterial agent, and *salmonella enteritidis* was used as the test strain, based on the liquid culture and plate culture system, combined with the determination of a series of physiological and biochemical indicators, from respiratory metabolism, energy metabolism, and material metabolism. To explore the effect of CEO on the physiological metabolism of

*S. enteritidis* to clarify the antibacterial mechanism of CEO on *S. enteritidis* and to provide a certain theoretical basis for the development of safe and efficient

*S. enteritidis* inhibitors. Studies had shown that the entry of bacteriostatic agents into cells may affect the physiological metabolism of cells, and various physiological metabolisms were closely related to life activities. Once inhibited, the bacteria will face the crisis of death (Dávila-Rodríguez et al., 2019; Hu et al., 2022). Therefore, this study explored the role of CEO on the physiological metabolism of *S. enteritidis* by measuring the protein synthesis ability, reducing sugar content, ATPase, and other related indicators of *S. enteritidis*, and providing a strong theoretical basis for further clarifying the antibacterial mechanism of CEO. This provides an important theoretical guidance and method for the inhibition of *S. enteritidis* contamination in the food industry.

## Materials and methods

### Materials and reagents

The cinnamon used in the present study was purchased from Baiweifu Food Co., Ltd. (Luoding, Guangdong, China). *Salmonella enteritidis* (ATCC BAA-664) was obtained from the China Microbial Culture Collection Center (Beijing, China). Resazurin was purchased from Shanghai Yuanye Biotechnology Co., Ltd (Shanghai, China). Acetone was purchased from Shanghai Bohr Chemical Reagent Co., Ltd (Shanghai, China). Malonic acid was purchased from Shanghai Zhongqin Chemical Reagent Co., Ltd (Shanghai, China). Iodoacetic acid was purchased from Shanghai Kejian Biotechnology Co., Ltd (Shanghai, China). Sodium phosphate was purchased from Shanghai Hengyuan Biochemical Reagent Co., Ltd (Shanghai, China). o-Nitrophenyl-β-D-galactoside was purchased from Shanghai Lianshuo Biotechnology Co., Ltd (Shanghai, China). All of the chemicals and solvents were of analytical grade. A H-1850R high-speed refrigerated centrifuge (Xiangyi Centrifuge Instrument Co., Ltd Changsha, China), UV-2550 spectrophotometer (Shimadzu Inc., Kyoto, Japan), SW-CJ-2FD ultraclean workbench (Antai Air Technology Co., Ltd, Suzhou, China), aDSX-280B portable pressure steam sterilizer (Shen'an Co., Ltd, Shanghai, China), Spectramax M2 Microplate Reader (MD Inc., USA), DHP-9080B intelligent constant temperature incubator (Langgan Experimental Equipment Co., Ltd, Shanghai, China), THZ-98AB thermostatic oscillator (Yiheng Scientific Instrument Co., Ltd, Shanghai, China), HQ30D dissolved oxygen meter (Yuyuelung Chemical Products Co., Ltd, Zhengzhou, China), JY92-IIN ultrasonic cell breaker (Xinzhi Biotechnology Co., Ltd, Ningbo, China), F-4700 fluorescence spectrophotometer (Kegu Technology Development Co., Ltd, Shanghai, China), and LC-10N-50A freeze drier (Lichen Technology Co., Ltd, Shaoxing, China) were used in our studies.

### Preparation of CEO

The appropriate amount of cinnamon peel was weighted and placed into a grinder to crush, and then through a 40-mesh sieve, the powder was collected and set aside. MarjanaRadünz's method for CEO extraction required 200 g of cinnamon powder to be placed in a 3000-ml round bottom flask, to which 2000 ml of distilled water was added, shaken thoroughly, heated, micro-boiled for the start point timing, and distilled for 4 h, to obtain the CEO (Payal et al., 2021; Radünz et al., 2021). This experiment analyzed the chemical composition of CEO through gas chromatography–mass spectrometry (GC-MS), and specific conditions and instruments of the setting parameters of the analyzer were as follows: (1) gas chromatographic conditions: chromatographic column DB-17MS(30 m × 250 mm, 0.25 m), program setup: the initial temperature was 50°C for 1 min, and the temperature first was raised to 220°C (4°C/min), held for 8 min, and then raised to 250°C (20°C/min), and held for 2 min. The injection temperature was 250°C, the helium carrier gas and the column flow were set to 1.0 ml/min, split flow injection and split flow ratio was 250:1. (2) Quality spectral conditions: the injection mode was electron impact (EI) ionization sub-source, electronic energy 70 eV, ion source temperature 250°C, interface the temperature was 230°C, and the scanning range of relative molecular weight was 15–500 u.

### Influence of CEO on the metabolic activity of *S. enteritidis*

#### Determination of metabolism

The various catabolism and anabolism in living cells were collectively referred to as metabolism. The *S. enteritidis* bacterial suspension in the logarithmic phase was taken and treated as metabolism. The *S. enteritidis* bacterial suspension in the logarithmic phase was taken and treated with 1/2 MIC (0.8 μL.mL^−1^) and MIC concentrations of CEO for 4 h, and a control group was set at the same time. Then, 10% resazurin was added and incubated at 37°C for 2 h on a shaker to make resazurin fully diffuse into the cells and centrifuged (4°C, 10,000 rpm for 4 min) to take the supernatant, then the fluorescence intensity was measured at 560 nm and 590 nm (Varçin et al., 2021).

#### Determination of cell membrane respiratory chain dehydrogenase

In the overnight cultured *S. enteritidis* bacterial suspension, different concentrations of CEO (1/2 MIC, MIC) were added to treat samples for 0, 2, 4, 6, and 8 h. In total, four test tubes were prepared, one test tube was boiled with the control sample in boiling water for 10 min, which was the negative control, and the other test tube was boiled with the control sample as the positive control. Approximately 1 ml aspirate of bacterial suspension was centrifuged to discard the supernatant, washed with phosphate buffer saline (PBS), added 0.9 ml of PBS to resuspend to OD_600_ of 0.5, then added 0.1 ml of 0.5% iodonitrotetrazolium chloride (INT) solution, reacted in the dark at 37°C for 2 h, added 50 μL of formaldehyde was collected by centrifugation(4°C, 10,000 rpm for 4 min), and a mixture of acetone and ethanol (1:1) was added, mixed, and centrifuged two times to combine the supernatant, and finally determined at OD_490_ (Yang et al., 2019).

#### Determination of cell metabolic viability

The method of Tapia et al. (2018) was referred to with slight modifications. Different concentrations of CEO (1/2 MIC and MIC) solutions were added to the bacterial suspension cultured to the logarithmic growth phase, and a control group was set. After treatment at 37°C for 2 h, the supernatant was discarded by centrifugation (4°C, 10,000 rpm for 4 min), washed with physiological saline, and resuspended, and then 1 mmol.L^−1^ INT was added and reacted at 37°C for 30 min. Finally, OD_630_ was measured. The experiment was repeated three times, and the results were averaged.

### Influence of CEO on the respiratory metabolism of *S. enteritidis*

#### Determination of initial respiratory rate (R_0_)

The oxygen consumption per time unit and mass unit of microorganisms were defined as the respiration rate. Therefore, the respiration rate of the bacteria could be judged by measuring the change of dissolved oxygen in the bacteria solution. The bacterial suspension in the logarithmic growth phase was obtained, 1, 0.4, and 3.6 ml of bacterial suspension, 1% glucose solution, and PBS solution were added, and the mixture was completely stirred within 5 min. The experiment was repeated three times, and the results were averaged (Consumi et al., 2020).


$$\begin{array}{l} R0=\\ \frac{\text{Dissolved oxygen meter reading}\left(\text{mg}/\text{L}\right)\times \text{Reactionvolume}\left(\text{mg}/\text{L}\right)}{\text{Reaction time }\left(\text{min}\right)\times \text{The total mass of bacteria in the reaction solution }\left(\text{g}\right)} \end{array}$$

(R_0_ : initial respiratory rate).

#### Determination of respiratory depression rate (IR)

According to the above-mentioned preparation steps of the initial reaction system, iodoacetic acid, malonic acid, sodium phosphate, and CEO of MIC concentration were added to four centrifuged tubes, stirred completely within 5 min, sealed, and stood; the dissolved oxygen content was measured, and R1 and IR were calculated. The experiment was repeated three times, and the results were averaged (Consumi et al., 2020).


$$\begin{array}{l} IR=\frac{R0-R1}{R0}\times 100 \end{array}$$

(IR: respiratory depression rate, R_1_ : dissolved oxygen, R_0_ : initial respiration rate).

#### Determination of respiratory superposition rate (DR)

Determining the overlap rate of typical inhibitors and CEO could roughly infer the main inhibitory pathways of CEO. The greater the overlap rate, the weaker the synergy between the two, and the smaller the overlap rate, the stronger the synergy between the two. According to the above preparation steps of bacterial suspension, CEO of MIC was added to three centrifuged tubes containing bacterial suspension, stirred well within 5 min, sealed, and stood; dissolved oxygen was measured, and R_1_ was calculated. Malonic acid, iodoacetic acid, and sodium phosphate were then added to each centrifuge tube, stirred, and mixed well. This was sealed until the system was stable; the dissolved oxygen R_2_ was measured, and DR was calculated (Zhang et al., 2020). The formulas are as follows:


$$\begin{array}{l} DR=\frac{R1-R2}{R1}\times 100 \end{array}$$

(DR: respiratory superposition rate, R_1_ : dissolved oxygen, R_2_ : dissolved oxygen).

#### Influence of CEO on key enzyme activities in the tricarboxylic acid cycle (TCA)

The above respiratory metabolism test results indicated that CEO mainly affected respiratory metabolism by inhibiting the TCA cycle of *S. enteritidis*. In the TCA cycle, ICDHm, CS, and α-KGDH were the three key enzymes. Therefore, the effect of CEO on the activities of these three key enzymes needs to be further explored. 1/2 MIC and MIC concentration of CEO were added to the bacterial suspension in the logarithmic phase, the samples were taken after 0, 2, 4, 6, and 8 h of shaking at 37°C, and set a control group at the same time. Finally, the instructions of ICDHm, CS, and α-KGDH kits (Jiangsu Jiancheng Bioengineering Institute; Li et al., 2022) were followed.

### Influence of CEO on the energy metabolism of *S. enteritidis*

#### Determination of ATP content

The method of Wang et al. (2019) was referred to for the determination of ATP content with slight modifications. *S. enteritidis* was inoculated in NB and cultured to logarithmic phase, and then, different concentrations (1/2 MIC, MIC) of CEO were added to the samples for 0, 2, 4, 6, and 8 h, and a control group was set at the same time and centrifuged at 5,328 *g* at low temperature. After 10 min, the supernatant was removed, washed with normal saline, and resuspended to an OD_600_ of 2.0. The supernatant was sonicated, centrifuged, and snap-frozen at −80°C for later use. The determination of ATP content was completed according to the instructions of the ATP content kit (LEYU Biotechnology, Shanghai).

#### Determination of ATPase activity

The pretreatment was the same as the operation method for ATP content, and the supernatant was taken for later use. The assay was done following the ATPase kit (Jiangsu Jiancheng Bioengineering Institute; Wang et al., 2019). The experiment was repeated three times and the results were averaged.

### Effect of CEO on substance metabolism of *S. enteritidis*

#### Determination of reducing sugar content

The content of reducing sugars was determined by the 3,5-dinitrosalicylic acid (DNS) method (Tiatira et al., 2022). First, the glucose standard curve equation obtained through the test is as follows: y = 0.524x−0.0187, and the correlation coefficient *R*^2^ = 0.9954. In the log phase bacterial suspension, CEO of 1/2 MIC and MIC concentration was added, a control group was set at the same time, and the samples were incubated at 37°C for 0, 2, 4, 6, and 8 h and centrifuged to obtain the supernatant. Approximately 0.5 ml of supernatant was removed, 1.5 m LDNS was added and treated with boiling water for 5 min, cooled, and then diluted with 20.0 ml of deionized water and measured OD540, and finally, the glucose content was calculated according to the standard curve equation obtained.

#### Intracellular protein content assay

Different concentrations (1/2 MIC and MIC) of CEO were added to the bacterial suspension in the logarithmic phase, treated for 4 h, and sampled centrifuged to discard the supernatant, and then, the precipitate was collected, washed three times with PBS, and resuspended. Then, the cells were disrupted using ultrasonic waves (JY92-ILN, 650W), and the pellets were discarded by centrifugation. The BCA protein quantification kit was used for the operation (Darmawati et al., 2019).

#### Determination of protein content by SDS-PAGE

The sample protein was extracted and took 1.08 μg/μL 50 μL added 5 μL buffer solution boiled for 5 min (denaturing the protein), cooled, and centrifuged at 6,000 rpm for 2 min, and the supernatant was taken to the sample loading. The prepared gel block was placed in the electrophoresis tank, and the inner and outer tanks were filled with electrode buffer solution to cover the entire sample adding hole. The processed samples were added into each sample adding hole in turn, the bacterial protein and marker were added to the hole with 12% separation gel, and then, the electrode was connected. The voltage was adjusted to 120kV for 1h, the glue plate was removed, stained for 1 h, destained several times, and finally, the results were recorded (Darmawati et al., 2019).

#### The effect of CEO on protein secondary structure

Referring to the method of Julian with slight modifications (Julian et al., 2022), the bacterial solution cultured to the logarithmic growth phase was added with different concentrations (1/2 MIC, MIC) of CEO for 4 h to the sample, while the control group was set. Then, the supernatant was discarded by centrifugation, washed three times with PBS, and placed in a Petri dish for vacuum freeze-drying. The dried samples were mixed with KBr, pressed into tablets, and finally scanned on an infrared spectrometer.

#### The effect of CEO on the fluorescence intensity of membrane proteins

According to the method of Nikan with slight modifications (Nikan et al., 2022), *S. enteritidis* was cultured at 37°C to the logarithmic growth phase, and then, the CEO of different concentrations (1/2 MIC, MIC) was added to continue the culture for 4 h, the control group was set at the same time, the supernatant was discarded by centrifugation, washed with PBS, and resuspended to an OD_620_ of approximately 0.4, and then, the fluorescence spectrum of the bacterial suspension was scanned within 300–500 nm.

#### Determination of β-galactosidase activity

Another important indicator for detecting cell membrane integrity was the activity of β-galactosidase. M9 lactose induction medium was added to the bacterial suspension in the log phase, after culturing for 10 h, different concentrations (1/2 MIC, MIC) of CEO were added to the treatment at 0, 2, 4, 6, and 8 h, and a control group was set at the same time. Then, the supernatant was discarded by centrifugation and an equal volume of TE buffer and lysozyme was added, mixed for 20 min, added to the reaction buffer of β-galactosidase, and then sonicated for 5 min. Finally, the supernatant was collected by centrifugation. ml of 1mLONPG and 2 ml of β-galactosidase buffer were mixed and reacted in a water bath at 37°C for 2 h, and the OD_405_ value was finally determined (Zeng et al., 2019).

### Data analysis

Data processing was performed using Microsoft Excel 2013; statistical analysis was performed using SPSS Statistics 25.0 software (*p* < 0.05); and plotting was performed by the Origin 2018 software.

## Results and analysis

### Influence of CEO on the metabolic activity of *S. enteritidis*

#### Determination of metabolism

**Figure 1:**
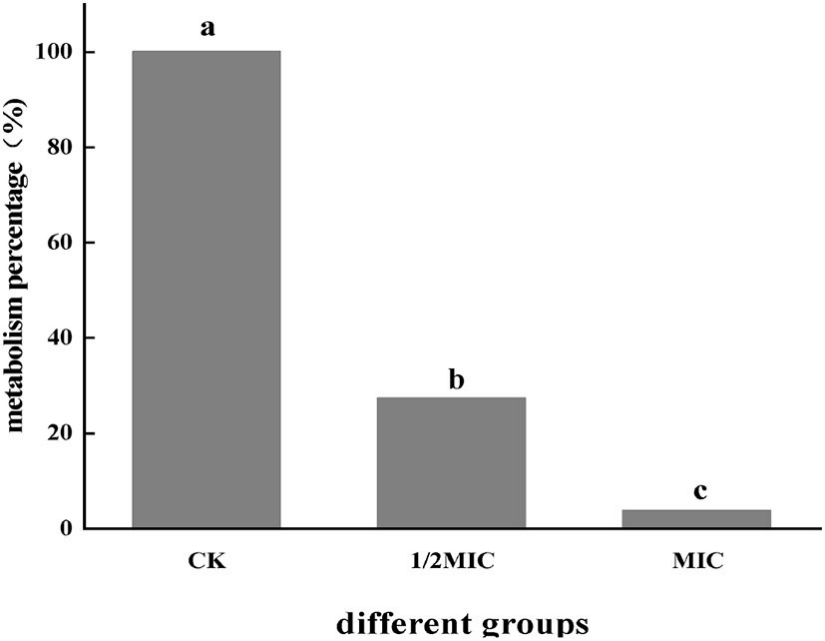
The effect of CEO on the metabolic capacity of *S. enteritidis*. (In the figure, CK represents the blank control group, MIC represents the minimum inhibitory concentration, and 1/2MIC represents half of the minimum inhibitory concentration), the same letter represents no significant difference between the groups, different letters represent significant differences (*p* < 0.05) between the groups.

As shown in Figure 1, After *S. enteritidis* was treated with CEO, the metabolic viability of the bacterium was determined using resazurin. The percentage of cells with metabolic capacity in the treatment group was compared to the control group. The percentage of metabolically active bacteria in the 1/2 MIC and MIC treatment groups accounted for 27.18% and 3.54% of the control group, respectively. This indicated that the addition of CEO significantly reduced the metabolic viability of the bacterium and laid the foundation for the subsequent investigation of the effect of CEO on the physiological metabolism of *S. enteritidis*.

#### The effect of CEO on the cell membrane respiratory chain dehydrogenase

**Figure 2:**
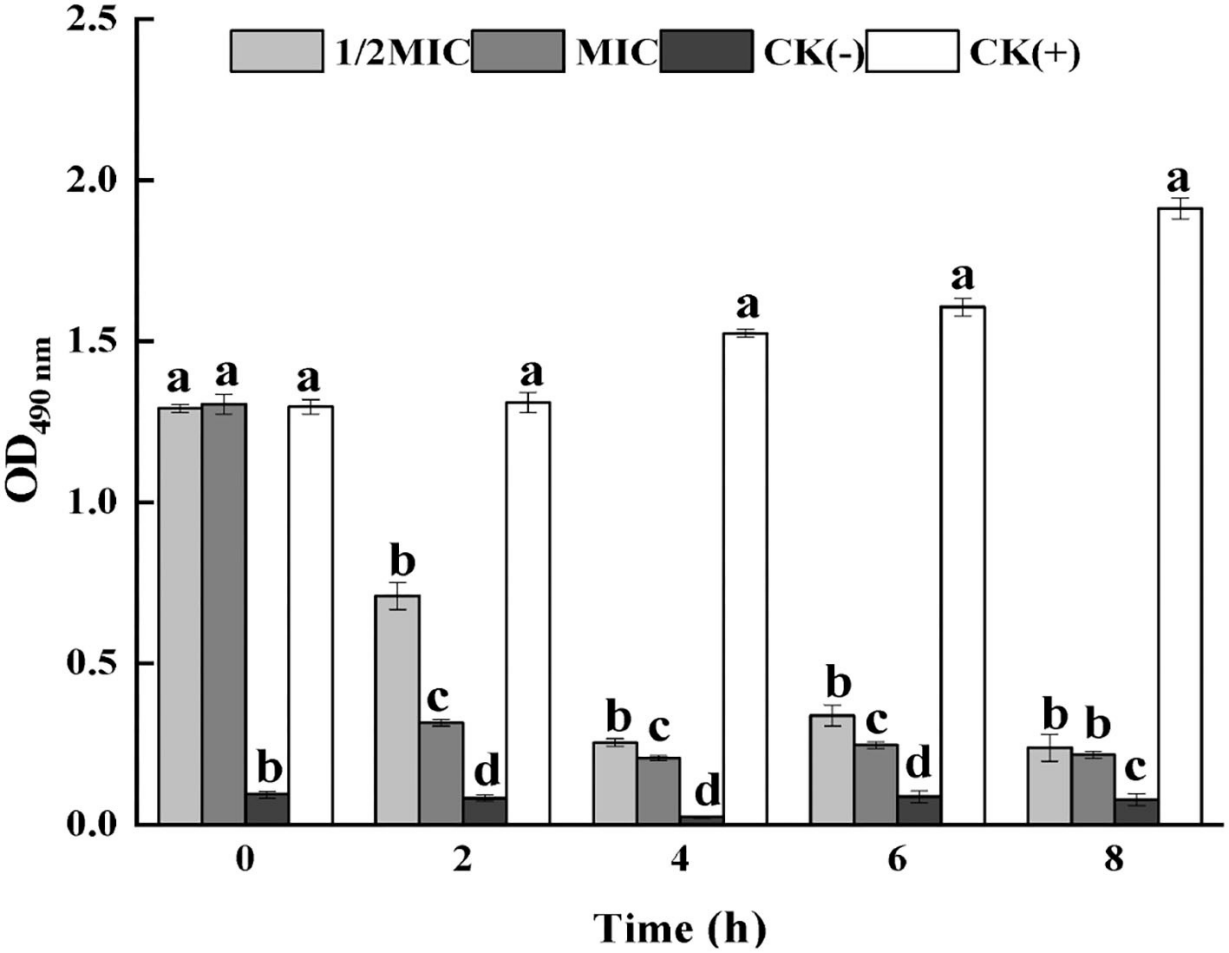
Inhibition of *S. enteritidis* respiratory chain dehydrogenase activity by CEO. (In the figure, CK represents the blank control group, MIC represents the minimum inhibitory concentration, and 1/2MIC represents half of the minimum inhibitory concentration, the same letter represents no significant difference between the groups, different letters represent significant differences (*p* < 0.05) between the groups).

Respiratory chain dehydrogenase activity is one of the important indicators of the metabolic activity of the bacterium. By measuring the effect of CEO on *S. enteritidis* cell membrane respiratory chain dehydrogenase, it could be seen from Figure 2 that compared with the positive control group, the respiratory chain dehydrogenase activity of the CEO treatment group was significantly reduced (*p* < 0.05), and the MIC treatment group is less active. It could be seen that CEO could inhibit the respiratory chain dehydrogenase activity of *S. enteritidis*, and the enzyme activity weakens with the increase of CEO concentration.

#### Influence of CEO on the metabolic viability of S. enteritidis cells

**Figure 3:**
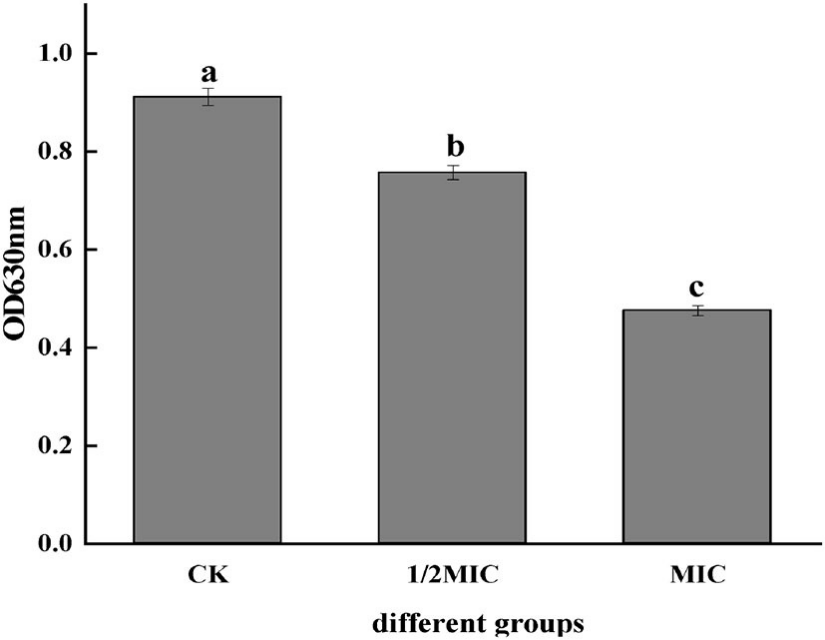
Effect of CEO on cell metabolism of *S. enteritidis*. (In the figure, CK represents the blank control group, MIC represents the minimum inhibitory concentration, and 1/2MIC represents half of the minimum inhibitory concentration, the same letter represents no significant difference between the groups, different letters represent significant differences (*p* < 0.05) between the groups).

The metabolic activity of bacterial cells could be determined according to the INT principle. The metabolic activity of the cell can be inferred by measuring the change of OD_630_ (Sun et al., 2018). It could be seen from Figure 3 that the OD_630_ of the CEO treatment group was significantly lower than that of the control group (*p* < 0.05) and showed a concentration-dependent effect, that was, the greater the CEO concentration, the lower the OD_630_. The OD_630_ value of the MIC group was 37.16% lower than that of the 1/2 MIC group, indicating that CEO could inhibit the metabolic activity of *S. enteritidis* cells.

### Influence of CEO on the respiratory metabolism of *S. enteritidis*

#### Determination of respiratory depression rate and superposition rate

**Figure 4:**
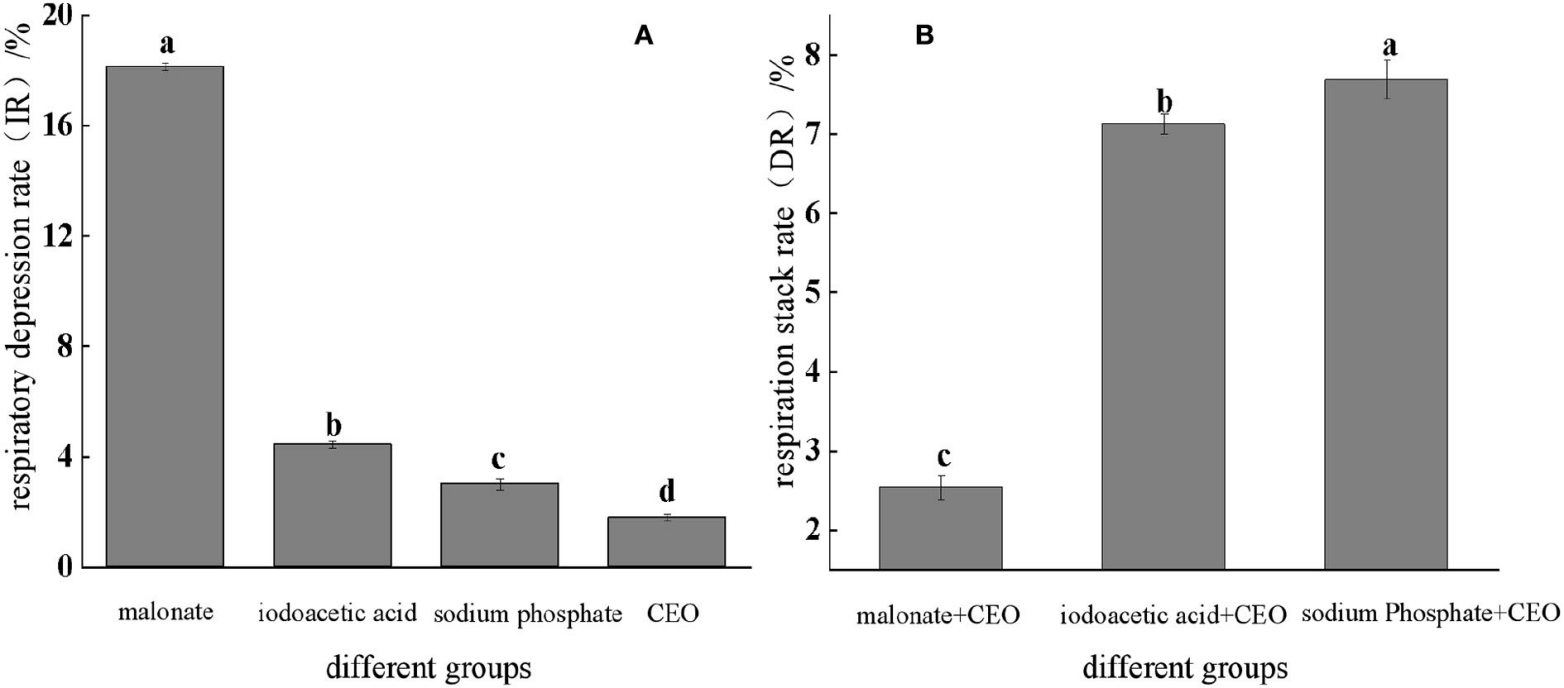
Respiratory inhibition and superposition of three typical inhibitors and CEO against *S. enteritidis*. (The same letter represents no significant difference between the groups, different letters represent significant differences (*p* < 0.05) between the groups).

As shown in Figure 4A, the respiratory depression rate was determined, and it was found that the CEO as well as three inhibitors had an effect on the respiratory metabolism of *S. enteritidis*, and the inhibition rate of malonic acid was significantly (*p* < 0.05) higher than the other two, which was 4.95%. With the respiratory stack rate, the main pathways were identified by which the CEO affected respiratory metabolism. It could be seen from Figure 4B that CEO and different respiratory inhibitors had a certain superposition effect, indicating that CEO had an inhibitory effect on the three production pathways of *S. enteritidis*, while the superposition rate of malonic acid and CEO was 2.49%, which was significantly lower than the other two groups (*p* < 0.05), indicating that the synergy between CEO and malonic acid was weak.

#### Determination of key enzyme activities in the TCA cycle

**Figure 5:**
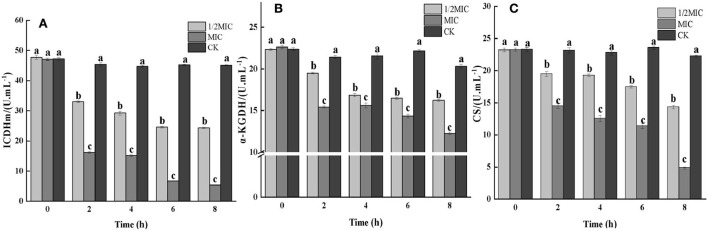
The effect of the CEO on the activities of key enzymes in the TCA cycle. (CK represents the blank control group, MIC represents the minimum inhibitory concentration, and 1/2MIC represents half of the minimum inhibitory concentration, the same letter represents no significant difference between the groups, different letters represent significant differences (*p* < 0.05) between the groups).

CS, ICDHm, and α-KGDH are three key enzymes in the TCA cycle. As shown in Figure 5, all three enzyme activities were decreased in the CEO-treated group compared to the control group. At 8 h, the enzymatic activities of ICDHm, α-KGDH, and CS in the 1/2 MIC treatment group were decreased by 47.99, 26.07, and 40.72%, respectively, compared with the control group. The enzymatic activities of ICDHm, α-KGDH, and CS in the MIC treatment group were reduced by 88.62, 45.23, and 79.14%, respectively. It could be seen that CEO had a significant inhibitory effect on the three key enzyme activities in the TCA cycle of *S. enteritidis* (*p* < 0.05), indicating that the CEO had a negative impact on the respiratory metabolism of *S. enteritidis*.

### The influence of CEO on the energy metabolism of *S. enteritidis*

#### Determination of ATP content

**Figure 6:**
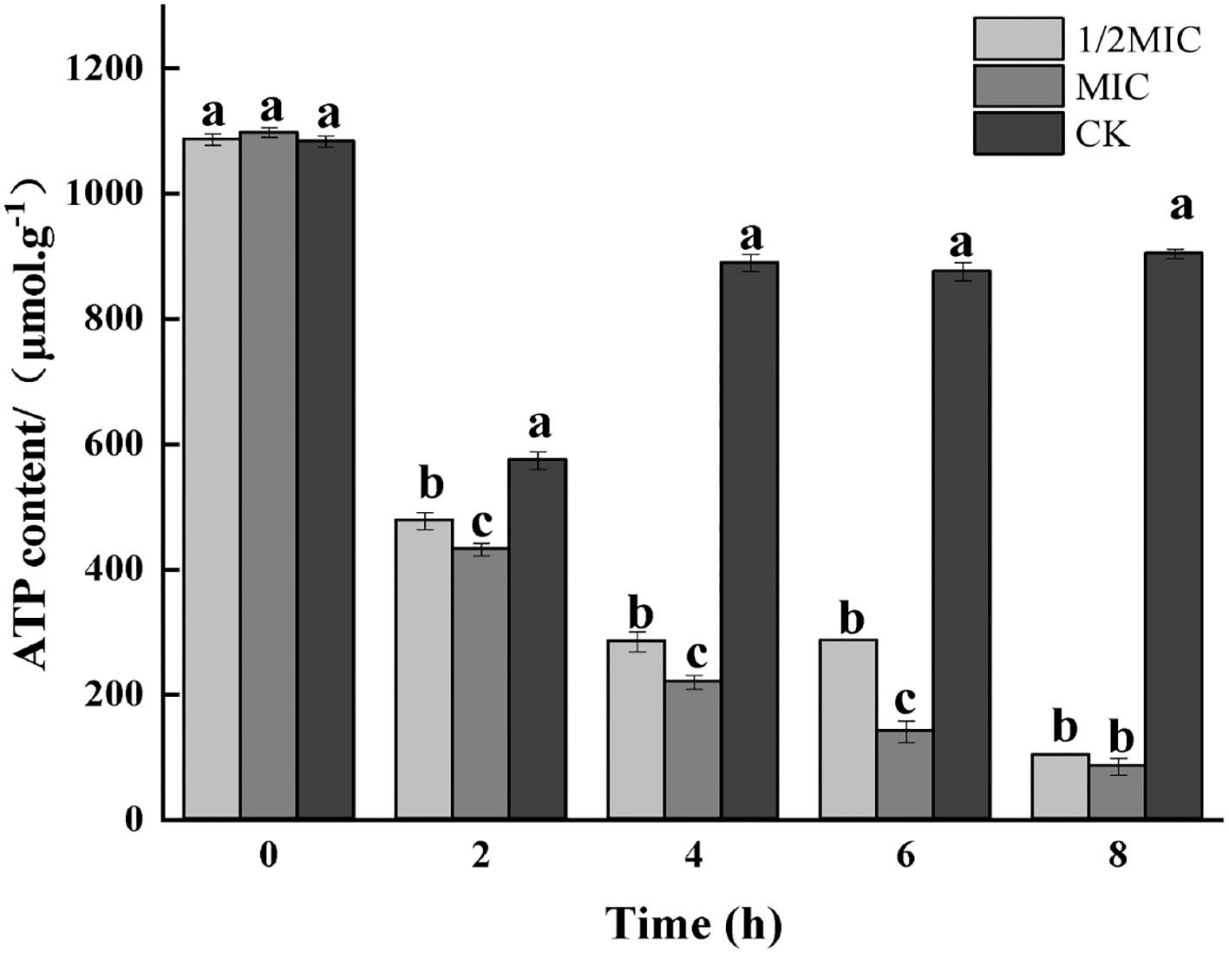
Effect of CEO treatment on the ATP content of *S. enteritidis*. (CK represents the blank control group, MIC represents the minimum inhibitory concentration, and 1/2MIC represents half of the minimum inhibitory concentration, the same letter represents no significant difference between the groups, different letters represent significant differences (*p* < 0.05) between the groups).

The existence of life was not only based on material metabolism but also driven by energy metabolism. ATP had the functions of acquiring, storing, and transporting energy, and its content changes were directly related to cellular energy metabolism. The cell membrane was responsible for the conversion of cellular energy, nutrient processing, the synthesis of structural macromolecules, and the secretion of various enzymes (He et al., 2022). The destruction of the bacterial cell membrane was closely related to the membrane-related energy conversion system. When the bacterial cell was damaged or had died, the ATP content would decrease, and the bacterial life activities would lose energy supply (Groves et al., 2018). The effect of the CEO on bacterial respiratory metabolism had been studied before, and this study further explores the effect of the CEO on ATP content. As shown in Figure 6, by measuring the ATP content of CEO on *S. enteritidis*-treated bacteria, it was found that the ATP content decreased in both treated and control groups at 2 h, and at 2 h, the ATP content of both the treatment group and the control group decreased. After 2 h, the ATP content of the treatment group decreased rapidly, while the control group still showed an increasing trend and stabilized. At 8 h, compared with the control group, the ATP content of 1/2 MIC and MIC treatment groups decreased to 88.74 and 90.02%, respectively. This indicated that CEO treatment would reduce the content of ATP in the bacteria, which might be due to the reduction of ATP synthesis and the increase in the rate of hydrolysis, thus resulting in insufficient energy required for bacterial life activities and ultimately inhibiting various metabolic physiological activities of the bacteria (Zeng et al., 2021).

#### Determination of ATPase

**Figure 7:**
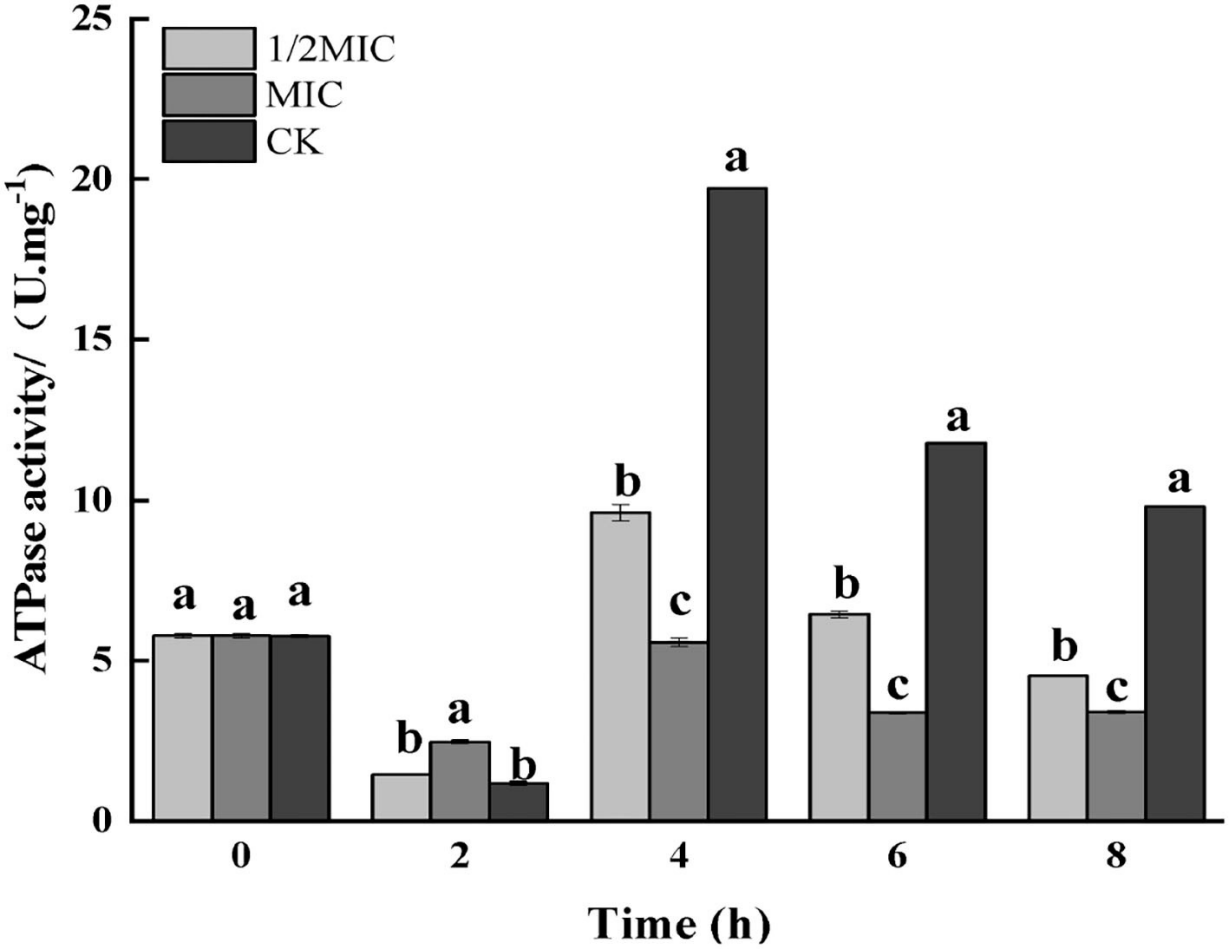
Effect on the enzyme activity of ATP in *S. enteritidis* by CEO. (The same letter represents no significant difference between the groups, different letters represent significant differences (*p* < 0.05) between the groups).

As shown in Figure 7, the results of the enzymatic activity of ATP showed that the enzymatic activity of ATP decreased in all treatment groups at 2 h, and the decrease in the control group was more significant than that in the treatment group (*p* < 0.05); after 4 h of treatment, the control group, 1/2 MIC, and MIC treatment groups were increased by 70.13, 36.78, and 0.51%, respectively, compared with the initial ATPase activity. After 4 h, the ATPase activity began to decrease again, and the decrease was more severe in the control group.

### The influence of CEO on the metabolism of *S. enteritidis*

#### Determination of reducing sugar content

**Figure 8:**
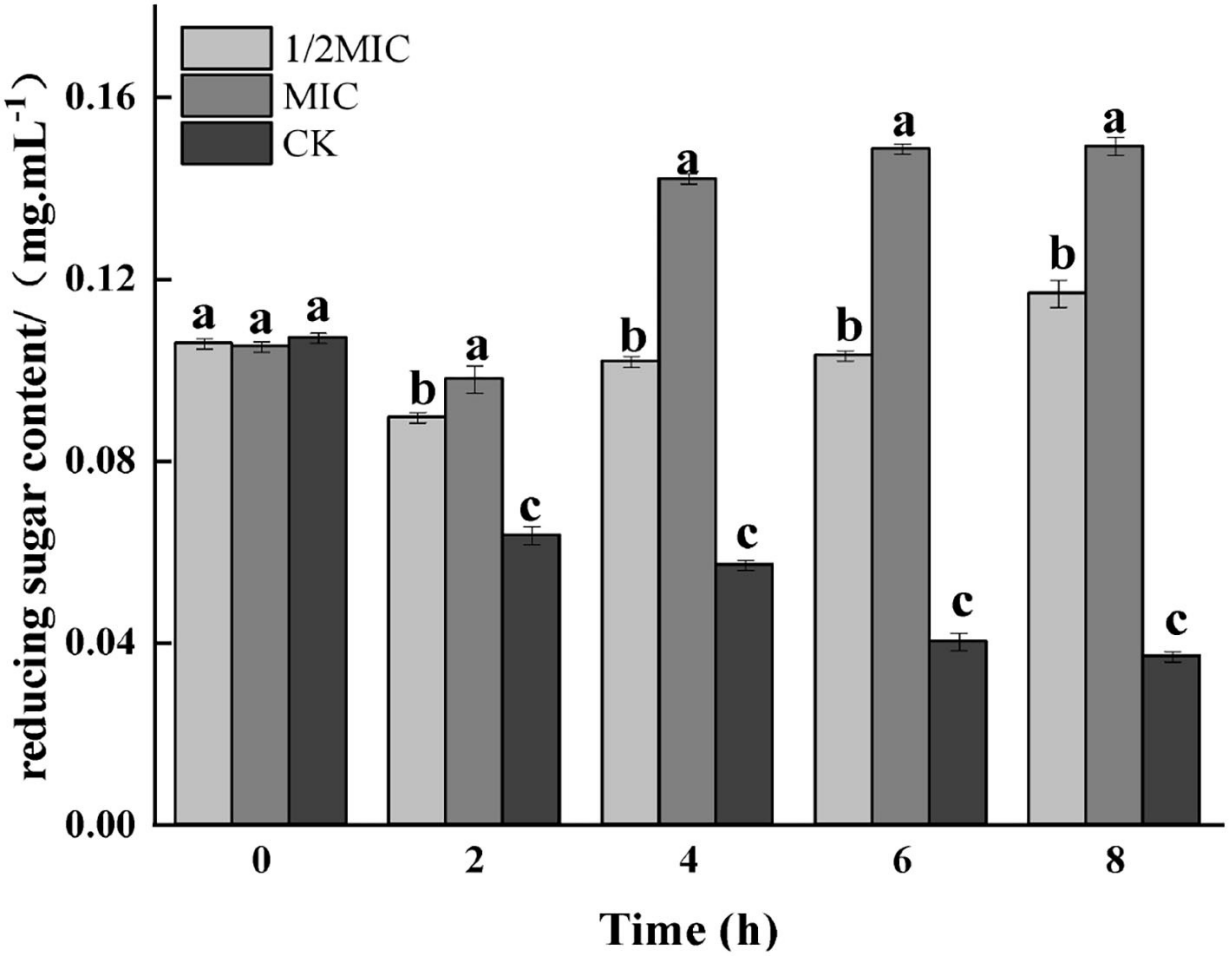
Effect on reducing sugar contents of medium for *S. enteritidis* by CEO. (The same letter represents no significant difference between the groups, different letters represent significant differences (*p* < 0.05) between the groups).

Carbohydrates were the key energy substances for bacteria to maintain life activities. The reducing sugar content not only presumed the structural integrity of the bacterium but also had an impact on the growth and metabolism of the bacterium. As shown in Figure 8, the reducing sugar content in the bacterial solution in the treatment group was significantly increased (*p* < 0.05), while an overall decreasing trend was observed in the control group. Compared with the control group, reducing sugar content in the treatment group decreased first and then increased. At 8 h, the 1/2 MIC treatment group was increased by 67.83%, while the MIC treatment group was increased by 74.83%.

#### The effect of CEO on intracellular protein content

**Figure 9:**
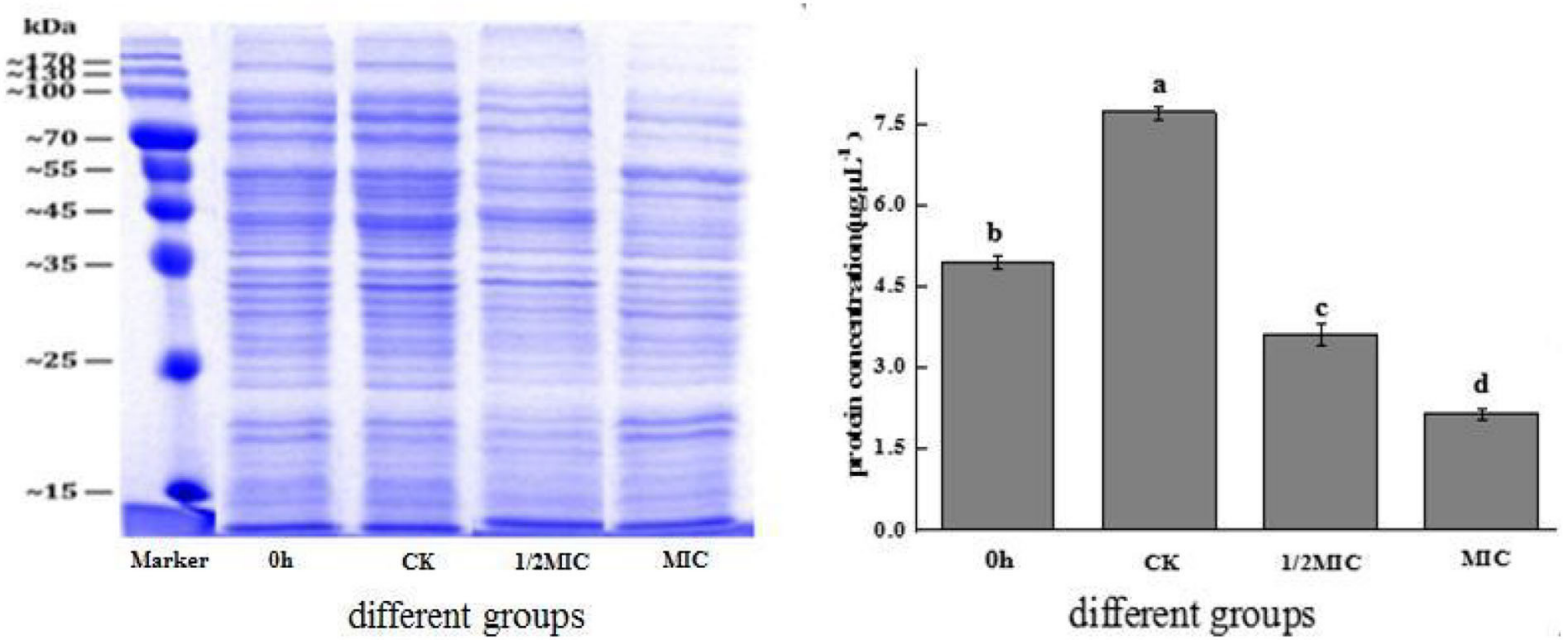
The effect of CEO on the total protein synthesis of *S. enteritidis*. (In the figure, CK represents the blank control group, MIC represents the minimum inhibitory concentration, 1/2MIC represents half of the minimum inhibitory concentration, and 0 h representative time is 0 h). The same letter represents no significant difference between the groups, different letters represent significant differences (*p* < 0.05) between the groups.

Protein was the necessary material basis to maintain the normal operation of bacterial life activities. It played an important role in the physiological metabolism of bacterial cells, and its spatial organization was also very important for cell reproduction (Yang H. et al., 2021). The results of SDS-PAGE electrophoresis are shown in Figure 9. The protein bands were well separated and clear, and the bands were mainly distributed in 15–170 kDa. The protein bands of the control group and the CEO-treated group were significantly different. At 4 h, compared with the control group, the color of the protein bands in the treatment group became lighter or disappeared, and the effect in the MIC group was more obvious, indicating that CEO treatment reduced the content of intracellular proteins. As could be seen from the figure, quantitative analysis by the BCA method found that compared with the control group, the 1/2 MIC and MIC treatment groups were reduced by 53.28 and 71.81%, respectively. The results showed that CEO treatment could significantly reduce the intracellular protein content of *S. enteritidis*. This result was consistent with that of sodium dodecyl sulfate-polyacrylamide gel electrophoresis (SDS-PAGE).

#### The influence of CEO on protein secondary structure

**Figure 10:**
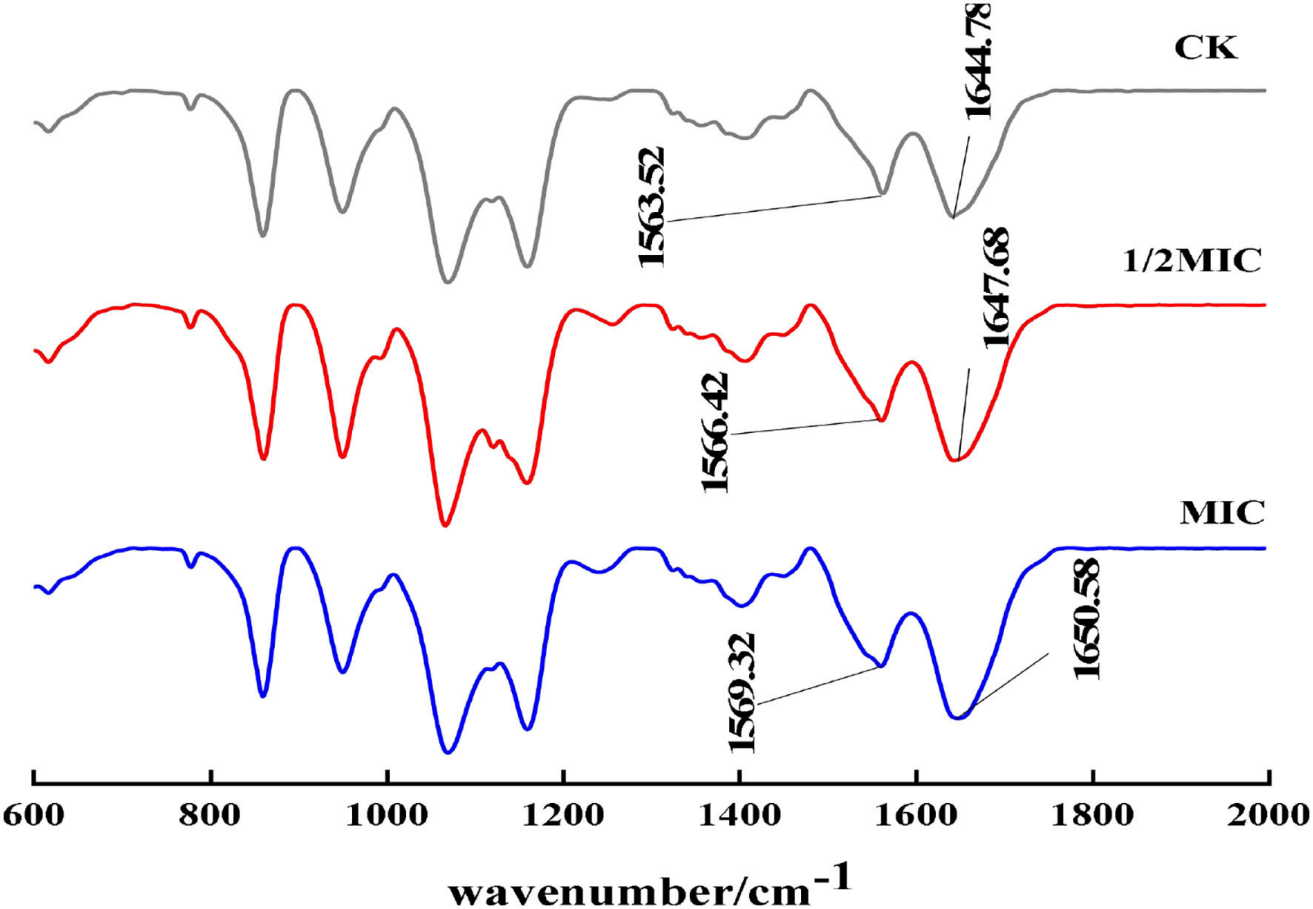
FT-IR spectra of *S. enteritidis* without and with CEO.

The most important infrared features to reflect the secondary structure of proteins were the amide I band and the amide II band. In particular, the amide I band could better reflect the secondary structure change of proteins. The amide I band was mainly the stretching vibration absorption of amino acid residue C=O. As shown in Figure 10, compared with the control group, the peaks of the amide I bands in the 1/2 MIC and MIC treatment groups moved from 1563.52 cm^−1^ to 1566.42 cm^−1^ and 1569.32 cm^−1^, respectively, and the peaks of the amide II bands, respectively, moved from 1644.78 cm^−1^ to 1647.68 cm^−1^ and 1650.58 cm^−1^, which indicated that the active molecule in CEO interacted with *S. enteritidis*, which caused the conformational change of the bacterial protein (Wu et al., 2018; Zhu et al., 2021).

#### The effect of CEO on membrane protein fluorescence intensity

**Figure 11:**
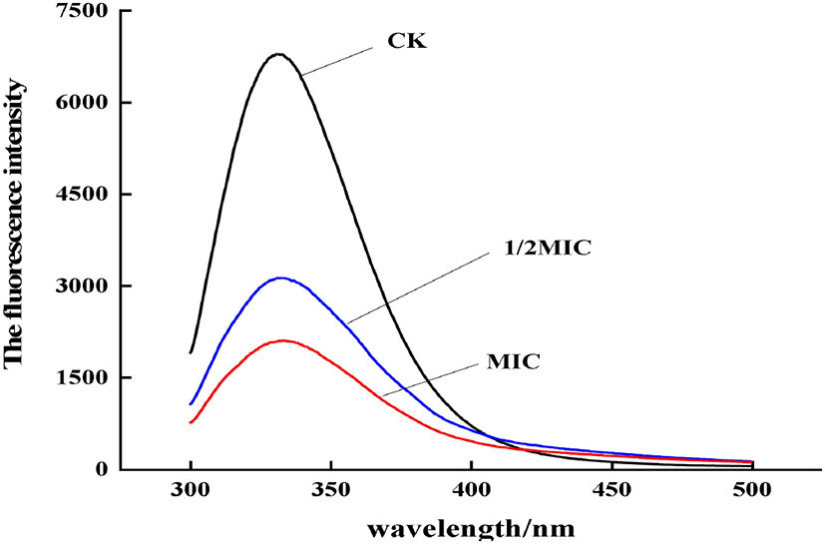
Fluorescence emission spectra of *S. enteritidis* treated by CEO with different concentration.

When the bacterial membrane protein was stimulated by antibacterial agents, the conformation would also change. As shown in Figure 11, by analyzing the change of endogenous fluorescence intensity in the bacterial solution, it was found that the maximum emission peak position did not change after CEO treatment, but the fluorescence intensity changed. Compared with the control group, the fluorescence intensity of the CEO treatment group was significantly lower, which might be due to the exposure to more phenylalanine, and phenylalanine contains an electron-withdrawing group –COOH, which led to the weakening of fluorescence; second, it might be that the CEO treatment made membrane proteins to coil or fold and made the exposed chromophore encapsulated by the macromolecular protein, thus resulting in the phenomenon of fluorescence quenching.

#### Determination of β-galactosidase activity

**Figure 12:**
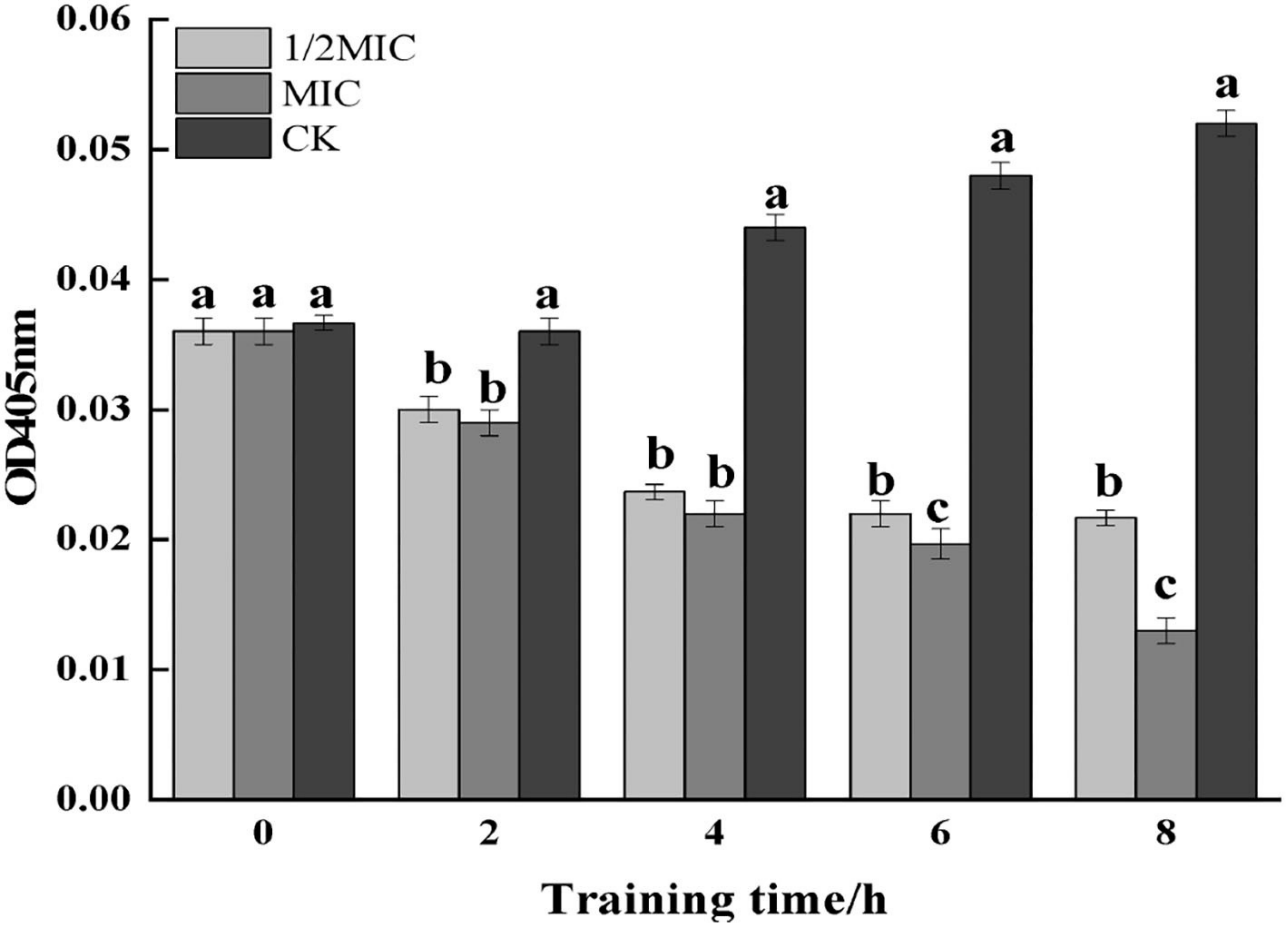
The effect of CEO on β-galactosidase activity of *S. enteritidis*. (In the figure, CK represents the blank control group, MIC represents the minimum inhibitory concentration, and 1/2MIC represents half of the minimum inhibitory concentration, the same letter represents no significant difference between the groups, different letters represent significant differences (*p* < 0.05) between the groups).

The β-galactosidase contained the cytoplasm of microorganisms such as bacteria could hydrolyze lactose, which was a key enzyme in bacterial physiological metabolism (Cui et al., 2021). As could be seen from Figure 12, the control group showed an upward trend, and compared with the control group, the treatment group showed a downward trend. Among them, the enzyme activity at 8 h was observed, and it was found that compared with the control group, the 1/2 MIC and MIC treatment groups were decreased by 58.59 and 74.53%, respectively. The results indicated that CEO had an inhibitory effect on the intracellular β-galactosidase activity of *S. enteritidis*.

## Discussion

In this study, we first determined the changes in the metabolic viability of *S. enteritidis* after CEO treatment and initially clarified that CEO had a certain inhibitory effect on the metabolic viability of *S. enteritidis* cells. To better understand the effects of CEO on the physiological metabolism of *S. enteritidis*, the effects of CEO on three aspects of respiratory metabolism, energy metabolism, and substance metabolism of *S. enteritidis* were further investigated.

The EMP pathway, the TCA cycle, and the HMP pathway were the three main pathways of respiratory energy production in the bacteriophage. In this study, the effects of three typical inhibitors and CEO on the respiratory depression rate and stack rate of *S. enteritidis* confirmed that CEO may affect the respiratory metabolism of the bacterium mainly by inhibiting the TCA cycle, thus cutting off the main energy source of the bacterium's life activity, which eventually led to the blockage of the bacterium's metabolism, and the key enzyme activities in the TCA cycle also produced significant inhibition. At the same time, CEO treatment decreased the ATP content in the bacterium, which may be a result of a decrease in the synthesis of ATP and an increase in the rate of hydrolysis, leading to a lack of energy required for the vital activities of the bacterium and ultimately inhibiting all metabolic physiological activities of the bacterium (Zeng et al., 2021). It can be seen that CEO has a good inhibitory effect on the physiological metabolism of *S. enteritidis*, which led to the disorder of physiological metabolism activities such as material transportation, energy, and information transmission of *S. enteritidis* and finally led to the loss of physiological functions and cell death. In 2005, HOLT and BARD also found that bacteriostatic agents could inhibit the respiration phenomenon of bacteria by measuring the change of dissolved oxygen concentration in bacterial liquid; the study found that mustard essential oil could destroy the cell membrane integrity of *Salmonella* (Yu Z. et al., 2019) reduced the content of ATP, and finally hindered the growth of the bacterial cells (Zhang et al., 2020), which was similar to this study.

The results of this study also showed that CEO inhibited the normal metabolism of proteins, and Han et al. (2020) also found that the bacteriostatic treatment reduced the protein band and the color of the bacteria, which was consistent with the conclusion of this study. Therefore, it was speculated that the CEO might hinder protein synthesis. CEO first destroyed the permeability barrier of the outer membrane of *S. enteritidis* and caused the loss of cell contents (Yu W. et al., 2019). Then, it entered the inner membrane and inhibited the activity of respiration-related enzymes, making the bacteria unable to perform normal respiration (Al-Nabulsi et al., 2020). It also acted on other components of the cell membrane, finally collapsing the cell membrane system (Huang et al., 2018). It then entered the cell to disrupt various physiological metabolic activities, resulting in bacterial lysis and death. A related study found that after treating common bacteria such as *Staphylococcus aureus* with bamboo leaf essential oil, the leakage of cell contents was aggravated, and the reducing sugar content in the bacterial suspension increased (Wang et al., 2020), which was consistent with the results of this study. Bao and others also found that the protein synthesis ability of *Salmonella* and other test bacteria treated with berberine from berberis serrata was weakened, and the activity of ATPase was reduced, which led to the disorder of normal physiological metabolism of the bacteria.

In addition, combined with the results of the previous study, the relative content of trans-cinnamaldehyde in CEO was high at 55.00%, and the remaining components were (–)-α- cubebene (13.28%), δ-cadinene (9.39%), α-clerene (5.22%), γ-mercyrrhine (2.11%), cuminol (1.61%), and cuminaldehyde (1.24%), and CEO showed a significant inhibitory effect on *S. enterica* and a significant concentration dependence, suggesting that CEO may have an inhibitory effect on *S. enterica* through the synergistic effect of multiple chemical components.

## Conclusion

In this study, by measuring the effect of CEO on the metabolism of *S. enteritidis*, it was found that CEO mainly inhibited the activities of key enzymes ICDHm, α-KGDH, and CS in the TCA cycle, ATP content, and ATPase activity, and CEO treatment decreased the protein content of *S. enteritidis*. Based on the research on respiratory metabolism, energy metabolism, and material metabolism, it was speculated that the antibacterial mechanism of CEO on *S. enteritidis* was to damage the permeability of the cell membrane, which led to the loss of the integrity of the cell membrane. Ultimately, bacterial growth was inhibited. This present work focused on the CEO's physiological metabolism of *S. enteritidis*, but there was minimal research at the molecular level. Therefore, further research on the key genes in the CEO's main regulatory system pathway of *S. enteritidis* as well as the CEO's antibacterial mechanism at the molecular level is required.

## Data availability statement

The original contributions presented in the study are included in the article/supplementary material, further inquiries can be directed to the corresponding author.

## Author contributions

ZZ: conceptualization, supervision, project administration, and funding acquisition. YZ: conceptualization, supervision, writing–reviewing and editing, project administration, and funding acquisition. XC: conceptualization, investigation, methodology, validation, and writing the original draft. LW, WeiL, WenL, JD, and SZ: validation. All authors contributed to the article and approved the submitted version.

## Funding

This work was supported by the National Key R&D Program (2019YFC1606500) and Gansu Agricultural University Science and Technology Innovation Fund (GAU-KYQD-2020-30).

## Conflict of interest

The authors declare that the research was conducted in the absence of any commercial or financial relationships that could be construed as a potential conflict of interest.

## Publisher's note

All claims expressed in this article are solely those of the authors and do not necessarily represent those of their affiliated organizations, or those of the publisher, the editors and the reviewers. Any product that may be evaluated in this article, or claim that may be made by its manufacturer, is not guaranteed or endorsed by the publisher.
